# Acrylamide in starchy foods subjected to deep-frying, 20 years after its discovery (2002-2022): a patent review

**DOI:** 10.12688/f1000research.140948.1

**Published:** 2023-10-12

**Authors:** William Yesid Díaz-Ávila, Sylvia María Villarreal-Archila, Francisco Javier Castellanos-Galeano

**Affiliations:** 1 Doctorate Program in Engineering-Faculty of Engineering-Agroindustrial processes group, Universidad de Caldas, Manizales, Caldas, 170001, Colombia; 2 Department of Industrial Engineering, Unidades Tecnologicas de Santander, Bucaramanga, Santander, 680005318, Colombia; 3 Department of Engineering, Center for Technology Development - Bioprocess and Agro-industry Plant, Universidad de Caldas, Manizales, Caldas, Colombia

**Keywords:** Asparaginase, Maillard reaction, Neoforms, Snacks, Starch

## Abstract

On the occasion of the 20th anniversary of the discovery of acrylamide in food, an analysis of patents related to the mitigation of this compound in food products obtained through immersion frying was carried out. For this purpose, a comprehensive search, compilation, and information analysis were conducted using free online databases such as Google Patents, Patenscope, and Lens. The search yielded a total of 79 patents within the considered time period (2002-2022). The countries with the highest number of granted patents were the United States, the European Union, and South Korea. The patents were classified into four main approaches: raw material modification (49%), application of pre-treatments (27%), process modification (16%), and measurement techniques (8%). Among the results, Frito-Lay, an American company, stands out as the food industry company with the highest number of granted patents, totaling 15. Based on this review, it is concluded that while a significant number of patents have been granted in recent years, there is still a lag in developing countries. Furthermore, more studies are needed to determine acrylamide in starchy food matrices subjected to immersion frying different from potatoes.

## Introduction

One of the dehydration processes that has become culturally and economically important is immersion frying. ^1^ In this process, food is immersed in hot oil, usually above the boiling point of water. ^2^ Its use is widespread in domestic and industrial settings due to its ease of operation, which gives the fried product unique characteristics of flavor, odor, color, and texture, making it very attractive to consumers. These changes are due to various phenomena: at the physical level, moisture loss and oil gain occur; at the rheological level, starch gelatinization occurs; at the chemical level, protein denaturation and non-enzymatic browning reactions, such as the Maillard reaction, occur. ^3^

This chain of reactions generates compounds responsible for fried products' characteristic aroma and color. However, multiple investigations have shown that toxic compounds can also be generated during this process, which depends on the nature of the raw material. ^4^^,^^5^ For example, high levels of heterocyclic amines and polycyclic aromatic hydrocarbons can be found in protein-rich foods such as beef, pork, and chicken. In the case of foods rich in carbohydrates or starchy matrices, significant levels of furans, hydroxymethylfurfural, and acrylamide have been detected. ^6^

Acrylamide is a white, odorless, crystalline substance highly soluble in water (2155 g/L at 30°C). It has a molecular weight of 71.08 g/mol, a melting point of 84.5°C, and a boiling point of 125°C at 25 mmHg. ^7^ This compound has been listed as potentially carcinogenic since 2002, when Swedish scientists discovered its presence in foods with high carbohydrate content and significant protein concentration, especially in roasting, baking, and frying processes where temperatures exceed 120°C. ^8^ Elevated levels of acrylamide have been found in potato chips, with values ranging from 330 to 2300 μg/kg, in corn flakes between 120 and 180 μg/kg, and in breakfast cereals, from almost undetectable levels to 1400 μg/kg. ^9^

The formation or synthesis of acrylamide in foods subjected to immersion frying might occur in different ways and by different means. The first is related to the interaction between a free amino acid, particularly asparagine, and a reducing sugar, such as glucose or fructose. ^10^ The second way in which this formation can occur corresponds to the interaction of the same amino acid with carbonyl group donor compounds other than reducing sugars, such as sugar fragments and lipids. ^11^ Finally, it can also occur due to acrolein, which originates from the thermooxidation of oils used in immersion frying. ^12^^,^^13^ These synthesis pathways require high temperatures and long processing times. ^14^

Research studies have identified that one of the parameters that can qualitatively indicate the presence of acrylamide in starchy matrices is related to the development of color and crust on the surface. ^15^ For example, in bread and French fries, an inverse correlation has been observed between product brightness and acrylamide level, indicating that as frying or baking time increases, the product tends to become more opaque and, therefore, the acrylamide level increases. As for crust formation, a direct relationship has been observed, showing that as the frying stage progresses, the product loses moisture and the proteins on the surface denature, which increases the thickness of the crust and the level of acrylamide. ^16^

Quantitative measurements of acrylamide in fried foods have been determined using techniques such as liquid chromatography coupled to tandem mass spectrometry (LC-MS/MS) ^17^ and gas chromatography coupled to mass spectrometry (GC-MS). ^18^ The extraction, identification, and quantification methods of food acrylamide levels have been developed and validated, although these methods are specific for each product type. ^19^ However, the high cost of the necessary equipment, materials, and reagents, ^20^ and the need for established protocols in factories dedicated to manufacturing these products have led to poor control in the measurement of this compound.

In countries, especially developing countries, there is a gap in the knowledge and application of measures for detecting and reducing acrylamide in starch matrices. However, regulations and guidelines have been implemented in developed countries to address this problem. The European Food Safety Authority (EFSA) established EU Regulation 2017/2158, which provides recommendations and regulations to mitigate acrylamide levels in foods such as coffee, potato chips, breakfast cereals, cookies, bakery products, and baby food. ^21^ Other countries have adopted the Codex Alimentarius Code on “practices for the reduction of acrylamide in food (CAC/RCP 67-2009),” which guides preventing and reducing acrylamide formation in potato and cereal products. ^22^ In the United States, the Food and Drug Administration (FDA) presented guidance in 2016 that describes strategies to reduce acrylamide formation, which is monitored by the Center for Food Safety and Applied Nutrition (CFSAN). ^23^

In the last 20 years, several ways to mitigate acrylamide in the food industry have been proposed. One of them consists of using raw materials with low acrylamide precursor content, such as reducing sugars and asparagine. Regarding process control, temperatures below 120 °C and short exposure times are recommended. In addition, feedstock pretreatments have been developed to reduce acrylamide precursor levels. ^24^^,^^25^ These forms of mitigation have led to the filing of inventions using patents for intellectual protection. Consequently, a review of patents related to the identification, quantification, and regulation of acrylamide in starchy matrices subjected to immersion frying is presented below, which may be of interest to determine the main innovations in this field and to assess their applicability in factories with capital limitations to invest in the management of this toxic compound.

## Methods

### Information searching stage

For the development of this phase, a search was performed in three worldwide patent collection databases: Google Patents, Patenscope and Lens Org. A search equation was designed using keywords, synonyms, and Boolean connectors such as “OR”, “AND”, and “AND NOT”.

The primary technology investigated focused on immersion frying processes, so the term “frying” with related English words (“fried” or “fryer”) was entered into the databases using the “OR” connector. Then, the term “acrylamide” was added to search for studies on acrylamide monitoring and reduction, delimiting the search string with the “AND" connector.

Subsequently, the search was refined to include only studies related to snacks. We also excluded raw materials not classified as starchy matrices, such as meat and fish words, using the Boolean negation “AND NOT” in Patenscope or the symbol “-” in Google Patents and Lens Org.

The results of the searches were recorded in a logbook, and the information was filtered, eliminating duplicate patent documents, and discarding those irrelevant to the work's objective, as well as patents in the process of being granted or abandoned.

### Information gathering stage

At this stage, the classification of patents was considered based on the country of origin where they have been granted, the year of concession, the most prominent applicant companies, and the different approaches that have been given to the area of acrylamide reduction and mitigation in the immersion frying process of starchy matrices.

### Information analysis stage

The documents were analyzed after collecting and organizing the information in an Excel database. Visualization diagrams were used to identify the main trends in monitoring and reducing acrylamide, the most common mitigation technologies and approaches, and the most active research areas. The number of granted patents was also determined based on inventors and major factories dedicated to producing starchy matrix snacks. Additionally, an analysis was conducted to determine which patent offices have granted the most patents in this field.

Finally, a critical review of the patent documents and associated annexes was performed, which provided detailed information on the inventions, methods, and technologies used to detect and reduce acrylamide in fried starchy matrix foods. This analysis provided a deeper understanding of specific advancements and proposed solutions in the field of immersion frying and starchy matrix snacks.

## Results and discussion

### Information search and gathering

The preliminary patent search allowed for the identification of the leading technologies applied by the industry related to monitoring and reducing acrylamide in starchy matrices subjected to the immersion frying process. The search equations were implemented in different databases, and the results are summarized in the logbook designed in Table 1.

**Table 1.** Summary of the search log for patent review.

Date	Datebase	Search span	Equation	Results
10/05/2022	Google Patents	2002-2022	(TI= frying) OR (TI= fried) OR (fryer) (acrylamide) (snack) -(meat OR fish)	105
11/07/2022	Google Patents	2002-2022	(TI= frying) OR (TI= fried) OR (fryer) (acrylamide) (snack) -(meat OR fish)	106
12/15/2022	Google Patents	2002-2022	(TI= frying) OR (TI= fried) OR (fryer) (acrylamide) (snack) -(meat OR fish)	107
10/05/2022	Patenscope	2002-2022	(frying OR fryer OR fried) AND acrylamide AND snack ANDNOT (meat OR fish)	280
11/07/2022	Patenscope	2002-2022	(frying OR fryer OR fried) AND acrylamide AND snack ANDNOT (meat OR fish)	280
12/15/2022	Patenscope	2002-2022	(frying OR fryer OR fried) AND acrylamide AND snack ANDNOT (meat OR fish)	282
10/05/2022	Lens Org	2002-2022	((frying OR fryer) AND (acrylamide AND snack)) - (meat OR fish)	95
11/07/2022	Lens Org	2002-2022	((frying OR fryer) AND (acrylamide AND snack)) - (meat OR fish)	96
12/15/2022	Lens Org	2002-2022	((frying OR fryer) AND (acrylamide AND snack)) - (meat OR fish)	97

**Figure 1.**
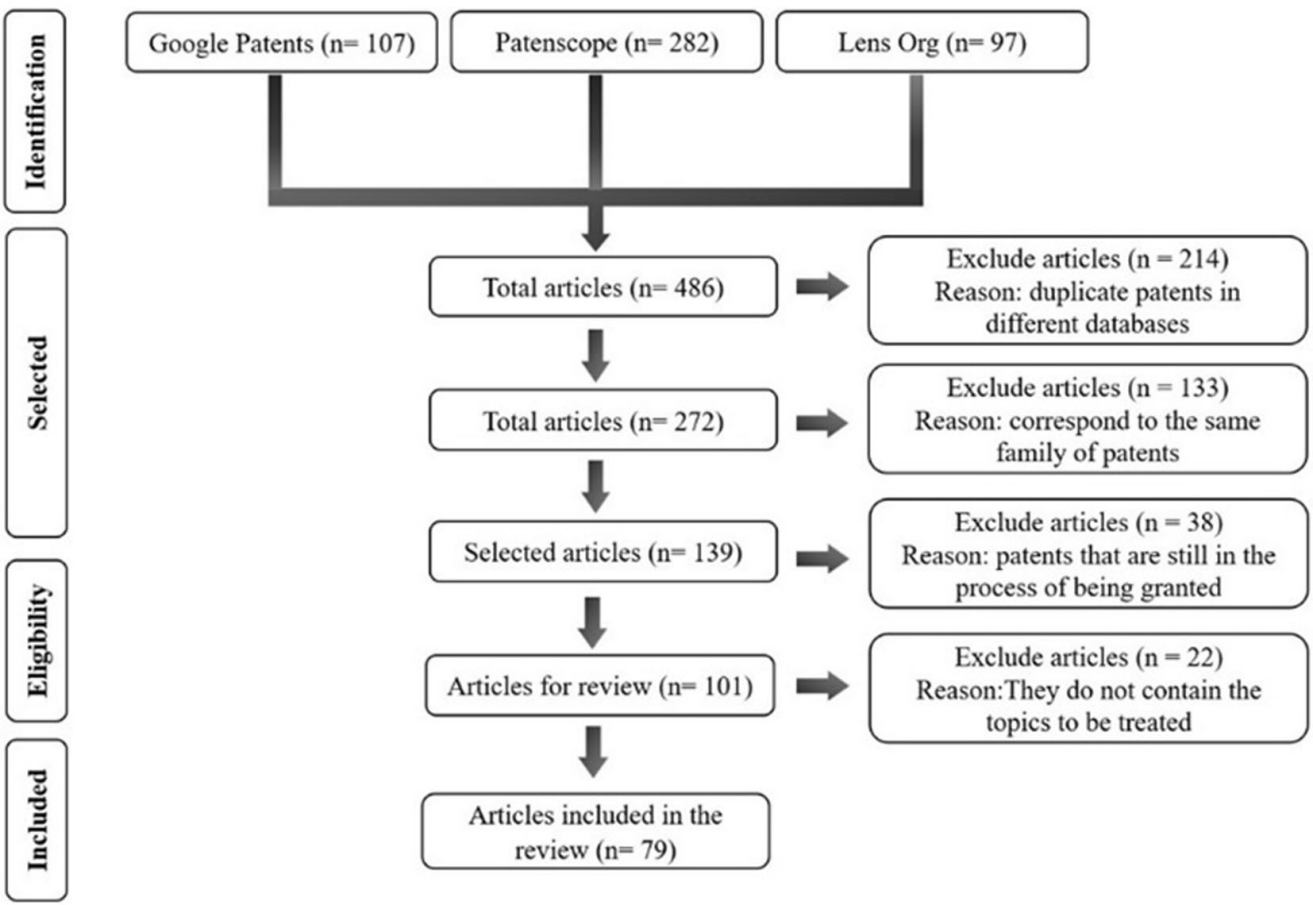
Process of selection and refinement of patents to include in the review.

Patenscope stood out as the database with the highest number of patents in this initial phase, with 282 documents. However, upon analyzing each document, it was observed that it includes the visualization of all patent families. The latter indicates that it refers to the same patent filed in different countries and years. On the other hand, Google Patents yielded 107 documents, and it was possible to refine the search by focusing on granted patents. Lens, meanwhile, provides additional information by allowing tracking of patents, such as the number of simple and extended families, and it displays the origin of the patent, marking the one designated as the priority. The number of obtained patents was refined by cross-referencing the information obtained from each of these search engines (see Figure 1). Patent documents unrelated to the study topic were excluded from the analysis.

It is important to note that patents were found whose claims and procedures did not address the immersion frying operation specifically, but rather the treatment of the raw material in these patents can be considered a preliminary stage to food frying. For example, in the patent developed by Haynes, Levine, ^26^ a method for developing stabilized wheat flour is proposed. During the milling process, amino acids can be released, which react with reducing sugars in the raw material and generate acrylamide due to the generated abrasion and heat. In this patent, the inventors suggest adding a portion of the coarse fraction of the grain to the fine fraction of the endosperm to avoid the presence of grits. With this method, it is possible to retain high levels of antioxidants and vitamins while substantially reducing acrylamide formation. This flour can be used to produce turnovers or breaded foods subjected to immersion frying.

### Country and year of grant

In 2002, Swedish researchers discovered that a potentially carcinogenic neurotoxic compound called acrylamide was formed during the thermal processing of food, mainly due to the interaction between reducing sugars and amino acids. Since then, numerous studies have been conducted to understand the effect of this compound on the body. Animal studies have shown that acrylamide causes DNA mutations, particularly in mice. However, definitive conclusions regarding its effects on humans have not been reached, and no minimum toxicity threshold has been established. It has been determined that the infant population is most exposed to acrylamide. For this reason, in countries belonging to the European Community, food factories have been requested to follow a series of recommendations and methodologies for monitoring and mitigating acrylamide in products such as potato snacks, coffee, cookies, and infant food.

**Figure 2.**
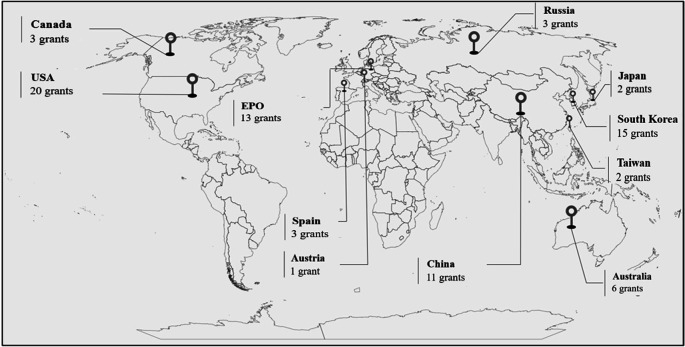
Number of patents granted by country, in the period 2002-2022.

The cartogram in Figure 2 details the leading patent offices that have granted patents on the control and mitigation of acrylamide. These include the United States (25.32%), the European Community (European Patent Office, Spain, and Austria) (21.52%), South Korea (18.99%), China (13.92%), and Australia (7.59%).

The wide range of fried products and their easy accessibility in fast-food restaurants have increased their global consumption worldwide. According to a recent survey, it is estimated that there will be a $208 billion increase in food sales in the United States across supermarkets, restaurants, fast-food establishments, and other retail food outlets by 2020. ^27^

In a globalized environment where competitiveness and business growth are based on entering new markets, companies must comply with the regulations issued by each target country for their diversification. That is why in countries such as China, South Korea, and Australia, among others, factories dedicated to snack production have shown interest in developing technologies to ensure that the fried products they market meet the highest quality standards, including compliance with the acrylamide levels established by European regulations.

**Figure 3.**
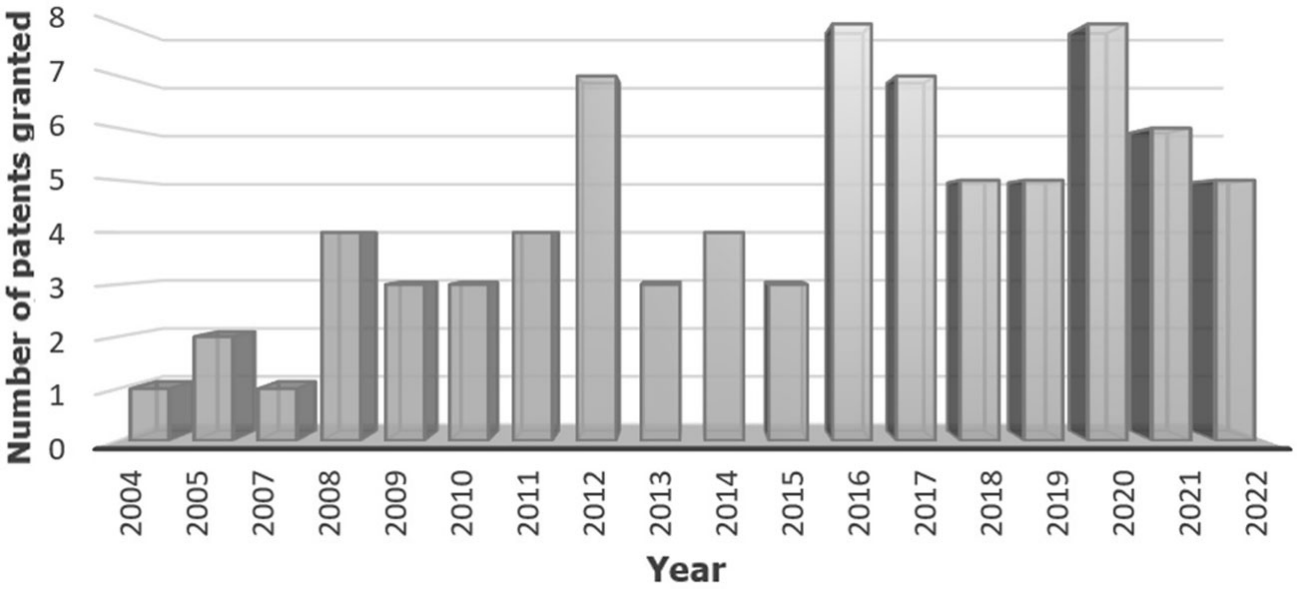
Number of patents granted per year in the period 2002-2022.

Figure 3 shows the number of patents issued yearly, revealing a growing trend in intellectual property protection for technologies and procedures to control and mitigate acrylamide in fried foods. Although the high level of acrylamide was discovered in 2002, the first patent related to this topic was granted in 2004. The European food and beverage industry, in collaboration with the European Commission, national authorities, and the scientific community at large, has invested significant resources in researching the carcinogenicity of acrylamide, aiming to understand its formation better and develop tools and techniques for its mitigation.

Due to the lack of a single solution to reduce acrylamide levels in the wide variety of foods where it has been found, the food industry has created documents with technical guidelines and steps to follow, applicable in commercial settings. One of these initiatives is the “Acrylamide Toolbox” document, first published in 2005 and developed by the association FoodDrinkEurope, which compiles the most comprehensive knowledge on the formation and potential mitigation of acrylamide in various foods. ^28^

Another key date in this timeline was the formulation of European Union Regulation EU 2017/2158. ^21^ An increase in the granting of patents can be observed from 2016 onwards. This increase can be attributed to the companies' interest in complying with the regulation that came into effect. The introduction of science and technology became fundamental pillars in this transformation process as companies sought to align with the established requirements.

### Classification and type of technology

**Figure 4.**
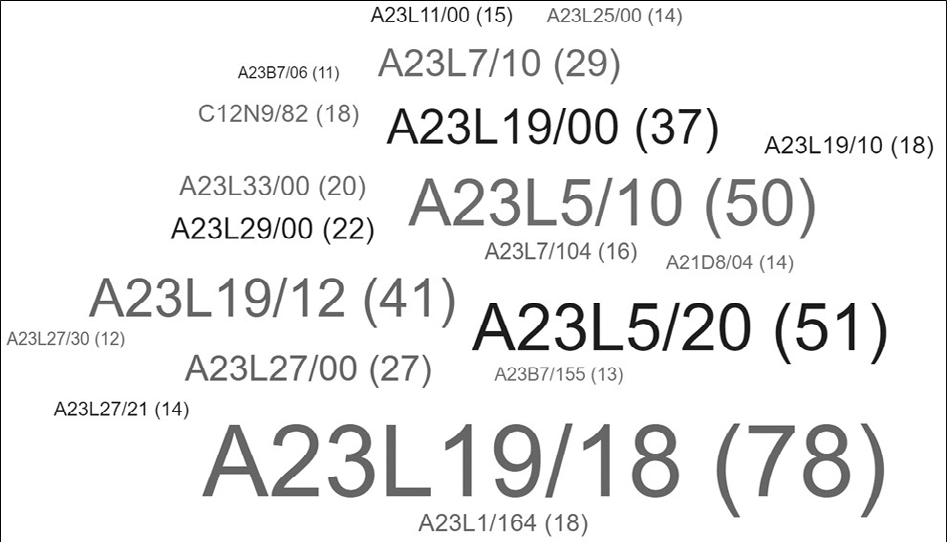
Patent classification according to IPCR.

The International Patent Classification (IPC), later reformed as IPCR, is based on the international multilateral treaty known as the Strasbourg Agreement, administered by the World Intellectual Property Organization (WIPO). Currently, 64 countries are party to this agreement. However, more than 100 industrial property offices, four regional offices, and the WIPO International Bureau use the IPCR under the Patent Cooperation Treaty (PCT) (OEPM, 2023). The IPCR classifies technology into eight sections that contain approximately 80,000 subdivisions. Each subdivision is represented by a symbol composed of Arabic numerals and letters of the Latin alphabet. ^29^ In this review, an analysis of patents was conducted, and a word cloud was generated, as summarized in Figure 4, with the main classification codes according to the IPCR, grouping patents according to the type of product and technology being implemented.

When examining the different classifications established in IPCR, ^30^ it can be observed that most of the patents shown in Figure 4 belong to the group of food or food products and their treatment (A23). Specific patents related to types of foods and processes can be observed within this subdivision. For the group of roasted or fried products, such as snacks or potato chips (A23L19/18), 78 patents have been assigned. For treatments involving removing unwanted matter, such as deodorization or detoxification (A23L5/20), 51 patents have been included. Additionally, 50 patents have been assigned to general methods of cooking foods, such as roasting or frying (A23L5/10). Furthermore, 41 patents on food products with potatoes as raw materials (A23L19/12) have been identified. In addition to these categories, a group of patents classified under group C, corresponding to the field of chemistry and metallurgy, can be observed. In this case, 18 patents related to the use of enzymes, such as asparaginase (C12N9/82), have been included.

The formation of acrylamide in starchy matrices is greatly influenced by the levels of its precursors, such as reducing sugars and asparagine. These precursors vary widely among plant varieties due to specific characteristics of each variety that regulate their biosynthesis, as well as factors such as soil, composition, climate, fertilizers, and storage conditions. Other factors that affect acrylamide formation are related to the recipe used (dilution and piece size, acidity), processing strategies (addition of exogenous additives, temperature, and humidity, pre-treatment), and preparation for consumption (baking, frying). ^31^

**Figure 5.**
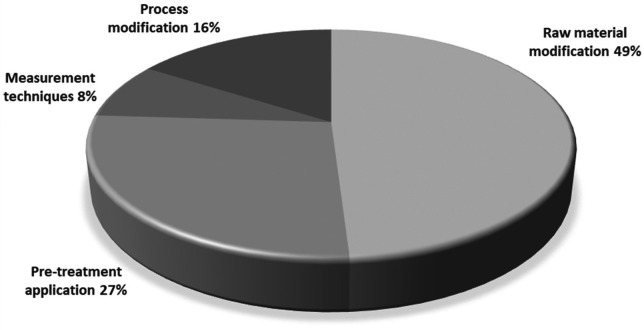
Distribution of total patents granted in the field of acrylamide monitoring and mitigation.

Based on these factors, various mitigation and inhibition strategies have been proposed to reduce acrylamide in model systems and real foods. According to the compilation of patents, four main thematic areas have been identified in which acrylamide monitoring and mitigation measures can be classified: raw material modification, pre-treatment application, process modification, and measurement techniques. Figure 5 presents the percentage distribution of the acrylamide monitoring and mitigation fields.

Raw material modification

It is evident that the highest percentage of patents related to acrylamide mitigation focuses on raw material modification (49%). Among the measures applied in this area, reducing precursors such as asparagine ^9^^,^^32^^–^^42^ and reducing sugars ^10^^,^^43^^,^^44^ are prominent. Inventors such as Budolfsen, Jensen ^25^ have proposed the simultaneous addition of asparaginases and enzymes capable of reacting with reducing sugars, such as oxidoreductases. These enzymes can be oxidases or dehydrogenases that react with glucose and maltose as substrates. The oxidase can be a glucose oxidase, a pyranose oxidase, a hexose oxidase, a galactose oxidase (EC 1.1.3.9), or a carbohydrate oxidase that exhibits higher activity on maltose than glucose. The concentrations and preparation of these enzymes to add them to the raw material are detailed in the claims of these patents.

From another perspective, inventors like Bhaskar and Topor ^45^ have considered the effect that enzyme application can have on consumer acceptance. To address this, they proposed the usage of raw materials with low native reducing sugar content (approximately 0.5%) and the application of a pre-treatment, such as blanching, to leach out these sugars. Subsequently, they add dextrose in an amount not exceeding 1%. This additive is low in reducing sugars and facilitates the Maillard reaction to achieve desired characteristics in the fried product with reduced acrylamide levels. Previously, Kim, Park ^46^ and Oku, Kubota ^47^ also utilized other sugar sources, such as trehalose, which maintains sensory characteristics of fried products with a significant decrease in formed acrylamide. During the development of a patented method to prevent acrylamide formation, Plank, Lewandowski ^48^ found that adding cyclodextrins (alpha, beta, and/or gamma and/or combinations thereof) to a food product or intermediate food can capture the amino acid asparagine through hydrophobic binding of the cyclodextrin, thereby preventing its reaction with a reducing end. Alternatively, cyclodextrin can sequester glucose or other small reducing sugars, avoiding interaction with free asparagine. The result is a reduction in acrylamide levels in the product.

Among other notable measures in raw material modification, the addition of natural antioxidants, ^2^^,^^6^^,^^49^^–^^55^ cationic salts, ^56^^–^^58^ vitamins, ^59^^,^^60^ organic acids, ^8^ amino acids, ^4^^,^^61^ and thiol sources such as N-acetyl-cysteine, dithiothreitol, casein, and combinations thereof ^62^ are highlighted. Vegetable oils are used as a heating medium in immersion frying processes. However, they are also considered part of the raw materials for fried products, as a portion of the oil is incorporated during the process. It is important for the oil to meet stability requirements and have adequate heat transfer properties, considering that its stability plays a significant role in acrylamide generation. Cao, Green ^63^ patented the use of oils with a high oleic acid content by genetically modifying safflower oilseed. The resulting oil exhibits good thermal resistance and provides high heat transfer rates, allowing for the production of crispy products with a satisfactory level of safety.

Pre-treatment application

The second line of importance corresponds to applying pre-treatments in starchy foods subjected to immersion frying, accounting for 27% of the results ( Figure 5). These pre-treatments include drying, ^64^^–^^66^ blanching, ^67^^,^^68^ electric pulses, ^69^ electroporation, ^70^ and others. ^3^^,^^71^^–^^78^ For example, inventors like Kim, Jeong ^79^ have patented the impregnation of amino acids such as arginine, valine, proline, sodium chloride, citric acid, and vitamin C, along with prior blanching and pre-frying treatments, for the production of potato snacks. On the other hand, Boudreaux, Desai ^80^ focus on a method to leach acrylamide precursors using an extract deficient in the precursors, proposing the leaching of asparagine and untreated potato starch with a potato extract lacking asparagine. Additionally, Barry, Burnham ^81^ and Sahagian ^82^ suggest that blanching can enhance the action of enzymes and the impregnation of salts and amino acids that compete with asparagine in the process of reducing acrylamide precursors. Moreover, Somsen and De Waele ^24^ point out that during blanching, in addition to leaching reducing sugars present in the raw materials, vitamins and minerals can also be lost, affecting the nutritional value, color, and flavor of the final fried products. Therefore, they propose performing consecutive washes with the blanching water and adding enzymes such as mannitol dehydrogenase and/or glucose-fructose oxidoreductase. This technique is known as “active blanching” and allows for removing acrylamide precursors and restoring minerals and vitamins lost during heating.

Process modification

Machinery and processing techniques are integral components in snack production, whether in domestic kitchens, commercial establishments, artisanal industries, or large-scale production units. These systems can vary in their level of sophistication, ranging from simple manual methods to automated plants capable of producing from a few kilograms to several tons of products per day. ^83^

Regarding modifications in the immersion frying process (16%), patents have been developed as a protective measure. One of these modifications is vacuum frying, ^84^^,^^85^ in which the system's pressure is adjusted, typically to pressures below atmospheric pressure. This results in a decrease in the boiling temperature of the water contained in the food, reducing the immersion time of the food in the oil, and decreasing the formation of acrylamide compared to frying under ambient conditions. However, it is noted that this technology may harm other quality attributes, such as the color and texture of the fried products.

To address this issue, Liu, Zhang ^86^ patented the use of a complementary color fixative in the vacuum frying process. This color fixative consists of a vacuum impregnator that coats the surface of the raw materials with Tartary buckwheat, gum Arabic, and L-tryptophan. The latter allows for the attainment of desirable sensory characteristics of fried products with reduced acrylamide levels. On the other hand, system pressures above atmospheric pressure have also been implemented. Johnson, Khan ^87^ designed an immersion frying apparatus that operates at pressures higher than atmospheric pressure, achieving low-oil-content potato snacks with a crispy texture. Their experiments obtained acrylamide levels below industry-acceptable standards and suggested that variations and modifications to the invention could be made without departing from its scope.

In addition to modifications in frying processes, patents have been developed that focus on improving the equipment used in the process, ^1^^,^^88^^–^^93^ aiming to increase thermal efficiency and ensure high standards of quality and safety in fried products. Among these inventions, the continuous frying system designed by Song, Kim ^14^ stands out, which utilizes a heating system and automatically controlled conveyor belts to regulate oil immersion temperatures and times, resulting in products with low acrylamide levels.

Another difficulty raised in immersion frying equipment is related to removing impurities generated during operation, which, due to reheating processes, can result in the formation of a significant amount of acrylamide that later migrates to the food. Kakita, Kojima ^94^ proposed a solution to this problem, especially in continuous frying equipment. They patented an adhesive cleaner that allows for the removal of particulate matter embedded in the conveyor belts. These patents include detailed plans and specifications of each of the components that make up the system, and their protection allows for replication only with the authorization of the inventors.

Measurements techniques

Measurement techniques for acrylamide have played a crucial role in implementing effective mitigation measures in industries, accounting for 8% of the patents found. Some approaches have utilized traditional direct measurement methods for acrylamide, ^95^ while other researchers have patented faster methodologies for real-time measurements. ^96^^,^^97^ Furthermore, measurements of other quality parameters have been linked to the acrylamide content in fried products. In the patent by Cantley, Desai, ^98^ intervention in the peeling and selection process of potato snacks based on coloration is proposed. This invention aims to achieve a crispy product through immersion frying, with an oil temperature between 146°C and 152°C, low moisture content (1.4%-2.0%), and removal of skins between 85% and 95%. The measurement of the color of the fried product is associated with the acrylamide content, which should be below 1000 ppb. This method has also been used to measure other quality characteristics of fried products, such as oil content. ^99^ Among the techniques developed for indirectly monitoring acrylamide, the patent developed by Hao, Liang, ^100^ stands out. They patented a method for measuring acidity in correlation with the level of polar compounds (PC) in the oils used in the immersion frying processes. There is a relationship between the formation of secondary compounds from lipid thermooxidation and acrylamide generation. Although it is not a direct method for detecting the level of acrylamide in fried foods, this technique can determine the point at which the oil is still considered suitable for use (PC≤27%), ensuring that the heating medium will not contribute to the formation of the toxic compound in the product.

### Applicants and inventors

**Figure 6.**
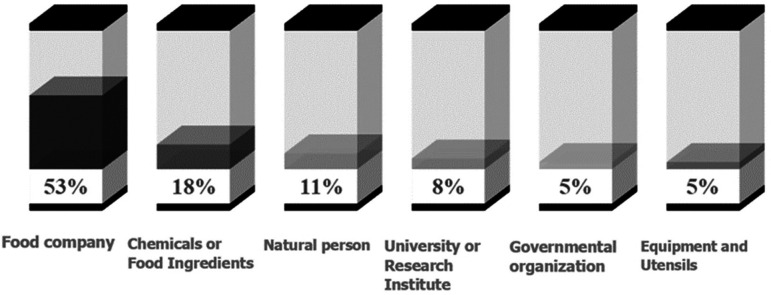
Classification of patent applicants related to acrylamide mitigation in immersion frying processes, over the past 20 years.

The industry seeks to adapt promptly to changes in consumer preferences, which involves developing products that demonstrate innovation and meet consumer expectations regarding functional or healthy snacks that offer greater nutritional value without compromising the characteristic taste and convenience. ^101^ In this context, the granting of patents related to controlling risks associated with immersion frying, particularly the regulation, and mitigation of acrylamide, can indicate these companies' commitment to producing nutritious and safe snacks. In Figure 6, patent applicants have been grouped into six categories based on their corporate identity and company description, who have filed patents related to the control and mitigation of acrylamide in the last 20 years.

Firstly, the food company sector (53%) is the most relevant group. Among these companies, Frito-Lay has associated 15 patents in the past 20 years, attributed to its parent company in the United States (Frito-Lay North America Inc.) and its subsidiary in Europe (Frito-Lay Trading Co GmbH). This company is part of the PepsiCo business group and produces some of the most popular and high-quality snacks in the United States and Canada, including Lay's and Ruffles potato chips, Doritos tortilla chips, Cheetos cheese-flavored snacks, Tostitos tortilla chips, Santitas tortilla chips, Sun Chips multigrain chips, and Fritos corn chips. Another relevant company is Procter & Gamble (P&G), with three patents. However, after selling Pringles to the Kellogg Company, P&G discontinued its product line, which included food and beverages. In Europe, snack factories such as Intersnack and Lorenz Snack-World from Germany are notable, as well as Molino Nicoli, an Italian producer offering gluten-free, allergen-free, non-genetically modified organisms (GMO), and organic snacks, breakfast cereals, and cereal bars. Leng-D'OR, a Spanish multinational dedicated to producing pellet-type snacks, is also prominent. In Asia, companies like Hoori Co., Ltd., specializing in manufacturing spiral-shaped potato snacks ("twist"), Nongshim, the largest producer of instant noodles and snacks in South Korea, Yamazaki Baking, the second-largest baking corporation in the world, and Xi'an Angel Food Co Ltd, a Chinese company that produces and distributes cakes, bread, mooncakes, among others, are noteworthy.

Secondly, factories and laboratories that produce chemicals or food ingredients (18%) are notable. Novozymes, a global biotechnology company based in Bagsværd, Denmark, stands out with four patents filed. Their main focus is on the use of enzymes and genetic improvement of enzymes, serving as suppliers to food factories. Zeracryl is a small Norwegian company that has developed a method to reduce the formation of the carcinogenic compound acrylamide during the industrial production of potato chips, either by using lactic acid bacteria or directly utilizing the metabolite. Emsland Group is an international German company that manufactures innovative products based on plant-based raw materials for the processing industry. They also offer a wide range of dried potato products under the brand “Mecklenburger Küche” for food retailers. Hayashibara Biochemical Laboratories Co Ltd develops, manufactures, and sells food ingredients, pharmaceuticals, cosmetics, dietary supplements, functional colorants, enzymes, and phospholipids.

In this analysis, nine individual inventors (11%) were also identified, who filed their patents as natural persons. Among them, inventors like Grune and Talarico, ^97^ designed a device and method for the rapid and reliable detection and determination of acrylamide concentration in food, as well as its prevention. Upon reviewing the impact of this patent, it was found that it has been cited 24 times in the design of other patents, demonstrating its technological relevance. Jeong ^59^ established a method for cooking potatoes with uniform holes to improve heat transfer to the interior and prevent the formation of harmful compounds. On the other hand, Lyutikov ^78^ designed a method for obtaining crispy cookies through the process of immersion frying, mixing different types of flour to achieve better organoleptic characteristics of the product. These inventions and others in the same group of patents ^2^^,^^51^^,^^54^^,^^66^^,^^84^^,^^90^ are mainly equipment and methodologies designed at a pilot scale in some cases scalable to an industrial level and do not require a large number of resources for their production.

According to Figure 6, universities and research institutes also play an important role in controlling and mitigating acrylamide in food (8%). In this aspect, the collaboration between universities and the food industry is highlighted, where the Industrial-Academic Cooperation Foundation of Konkuk University and the University-Industry Cooperation Foundation of Dankook University are key pillars for industrial development in their respective countries. The latter is reflected in patenting acrylamide mitigation mechanisms applicable to fried products. Additionally, the Institute of Food Science and Technology of the Chinese Academy of Agricultural Sciences (CAAS) and the Agrarian Technological Institute of Castilla and León (Itacyl) are notable organizations in comprehensive agronomic research, responsible for conducting basic and applied research activities, as well as developing new technologies with impacts in agriculture.

**Figure 7.**
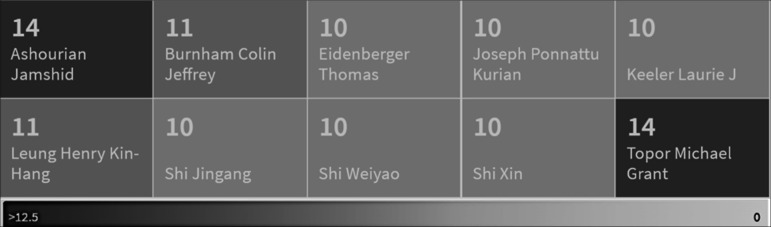
Top Inventors in the last twenty years.

In addition to universities and research institutes, some government-associated organizations (4%) and companies dedicated to manufacturing equipment and utensils (4%) have also been involved in designing inventions for acrylamide control. The Commonwealth Scientific and Industrial Research Organisation (CSIRO) of Australia has obtained a patent related to the control of oleic acids in oils, which helps extend their shelf life and prevent the formation of harmful compounds during frying processes. ^63^ Other government entities, such as the Korea Food Research Institute (KFRI), the Shanghai Chongming District Food and Drug Safety Committee Office, and the Korea Food Industry Cluster Promotion Agency, have also obtained patents related to acrylamide control. Regarding companies dedicated to manufacturing equipment and utensils, companies such as Nitoms, Semyung Total Co., Ltd., Lishui Yifanjia Mould Technology Co Ltd, and Chongqing Chengyue Food Co have obtained patents for designing more efficient equipment for producing safe fried products. Furthermore, the participation of inventors associated with the business environment in protecting their inventions is noteworthy ( Figure 7). Among the key inventors are Jamshid Ashourian, president and founder of Jimmyash Llc, and Michael Grant Topor, who have obtained 14 patents each. Colin J. Burnham, who has collaborated with the snack factory Fritolay, has patented 11 inventions. Dr. Thomas Eidenberger from the University of Applied Sciences Upper Austria, Joseph Ponnattu, Laurie Keeler, Jingang Shi, Weiyao Shi, and Xin Shi have each distinguished themselves with ten patents in the last 20 years.

## Conclusions

The study of patented technologies, treatments, and procedures for controlling and regulating acrylamide over the last 20 years has revealed that countries with established regulations in acrylamide generation in fried products have a higher number of granted patents. Additionally, there is a lag in the food industry of developing countries, where a lack of innovation is evident due to the scarce or nonexistent number of patents in the field of acrylamide mitigation.

The publication of EU Regulation 2017/2158 is considered an important date, as it has led to an increase in the trend of the number of granted patents during the study period. The classification conducted according to IPCR revealed that most patents (78) were related to fried products or snacks, demonstrating the effectiveness of the search equation used in different databases. The analysis of the various stages in which intervention can be made in the immersion frying process allowed for the identification that inventors have primarily focused their efforts on intervention in starchy raw materials, either through the addition of enzymes, antioxidants, or other compounds (49%), or through the implementation of pre-treatments such as drying, blanching, and electric pulses, among others (27%).

Vacuum frying, initially considered an alternative to atmospheric frying to obtain low-oil-content products, has also proven to be a viable technology for producing snacks with low acrylamide content. Fried product manufacturers have focused on automating their processes, patenting increasingly sophisticated equipment that allows for controlling variables such as temperature, pressure, and immersion time of the food in the oil. While academia has developed reliable measurement techniques, such as gas chromatography coupled with mass spectrometry, large companies have shown interest in developing faster measurement methods, either through direct measurement, the use of biosensors, or the evaluation of quality parameters that correlate with acrylamide levels, such as surface color. Among the leading factories that have protected inventions related to acrylamide control, Frito-Lay stands out, contributing to its global consolidation by complying with regulatory requirements in each country where it operates and meeting consumer demands. In general, it is concluded that although snack food factories have taken measures for acrylamide control and reduction, further advancements in this field are still required. Despite the numerous studies related to potatoes, it is necessary to determine the effectiveness of these measures in other starchy matrices.

On the other hand, integrating the analysis of patent applications is recommended, as they mark future trends in acrylamide mitigation measures. An increase in the number of applications can indicate a strengthening of technological development driven by market-oriented approaches based on new technologies. Lastly, a decrease in the number of applications may signal a shift in research and development priorities or resource limitations, such as the availability of development personnel and the costs associated with acquiring intellectual property. By predicting and understanding the background of these trends, it is possible to envision future products, technological plans, and business strategies in the field of acrylamide control and mitigation.

## Data Availability

Researchgate: Acrylamide Patent Analysis Database, https://doi.org/10.13140/RG.2.2.25415.47521. ^102^ This project contains the following underlying data:
• Acrylamide Patent Analysis Database.xlsx (Article analysis database: Acrylamide in starchy foods subjected to deep-frying, 20 years after its discovery (2002-2022): A patent review). Data are available under the terms of the Creative Commons Attribution 4.0 International license (CC-BY 4.0). ResearchGate: PRISMA checklist for “Acrylamide in starchy foods subjected to deep-frying, 20 years after its discovery (2002-2022): A patent review”, https://doi.org/10.13140/RG.2.2.33541.93927. ^103^ Data are available under the terms of the Creative Commons Attribution 4.0 International license (CC-BY 4.0).
