# Supplementary material for: Acrylamide in starchy foods subjected to deep-frying, 20 years after its discovery (2002-2022): a patent review
Source: F1000Res. 2024 Apr 19;12:1322. Originally published 2023 Oct 12. [Version 2] doi: 10.12688/f1000research.140948.2 (PMC10904932; doi:10.12688/f1000research.140948.2)
Supplement: Peer review history

# Reviewer report: Reviewer response for version 1

**DOI:** 10.5256/f1000research.154356.r234853

**Published:** 2024-02-14

**Authors:** Maria Alessia Schouten

**Affiliations:** 1 University of Bologna, Bologna, Italy

The aim of the introduction is to familiarise the reader with the challenges posed by the formation in starchy matrices during immersion frying. The introduction sets the stage for the subsequent examination of patents related to the identification, quantification and regulation of acrylamide and shows that the focus is on innovation and its applicability in factories with limited capital. The introduction is concise and sets the context for the examination of patents related to acrylamide in starchy matrices subjected to immersion frying. The stated aim is consistent with the subsequent sections and provides a clear and logical flow for the reader.

 The methods section accurately and clearly describes the systematic process of searching, collecting and analysing patent information related to acrylamide reduction in dip frying of starchy matrices. The methodology described is in line with best practice in patent searches and ensures a comprehensive and rigorous approach to data collection and analysis.

 The results and discussion illustrate the multi-faceted approach being taken globally to tackle acrylamide in fried starchy foods. The patents cover different stages of the food production process, which emphasises the importance of a comprehensive strategy. The collaboration between industry, research organisations and regulatory authorities underlines the commitment to food safety and quality. The findings provide valuable insights into the evolving landscape of acrylamide mitigation and offer a comprehensive understanding of technological advances and regulatory actions in the food industry over the past two decades.

 In summary, the conclusion provides a comprehensive review of the current state of acrylamide control technologies and draws attention to regulatory influences, global trends, industry practices and the potential for future innovation. It serves as a valuable resource for policymakers, industry professionals and researchers seeking to navigate the complex landscape of food safety and technology.

Are the rationale for, and objectives of, the Systematic Review clearly stated?

Yes

Is the statistical analysis and its interpretation appropriate?

Not applicable

Are sufficient details of the methods and analysis provided to allow replication by others?

Yes

Are the conclusions drawn adequately supported by the results presented in the review?

Yes

Reviewer Expertise:

Food technology

I confirm that I have read this submission and believe that I have an appropriate level of expertise to confirm that it is of an acceptable scientific standard.

# Reviewer report: Reviewer response for version 1

**DOI:** 10.5256/f1000research.154356.r234854

**Published:** 2024-02-14

**Authors:** Owen M. McDougal

**Affiliations:** 1 Boise State University, Boise, Idaho, USA

The manuscript is a good summary of the patents related to starchy foods, mainly potato products exposed to immersion frying. The content is well described and organized in terms of patents and the advancement of knowledge related to acrylamide in the past 20 years.

 Some notes:

1. Some citations don’t match the references.
2. Keywords include “neoforms”, which was not described or mentioned within the manuscript.
3. The introduction mentions that toxic compounds can be generated during immersion frying, however, the authors only mention the dependency on the nature of the raw material when several other factors can generate the formation of acrylamide.
4. There is no specific mention of the acrylamide limits determined by the EFSA, Codex Alimentarius, or FDA that can be used as a baseline for the patents mentioned in the results section.
5. There is a sentence in the results section that mentions a prediction of the estimated increase in food sales that is outdated. Also, the manuscript mentions developing countries, so an amount that describes the consumption in those developing countries and that can be tied to the need for acrylamide monitoring would be helpful.
6. In the classification section, a brief description of the classification group A21D8 would be useful.
7. In the pre-treatment application and process modification section, there is a description for the application of vacuum frying, but not for drying, blanching, electric pulses, or electroporation related to the patents mentioned.

The measurement techniques section is very brief and is missing detection methods like Computer-based visualization, spectrophotometry, atomic absorption, fluorescence, NIR, Raman spectroscopy, to mention a few.

Are the rationale for, and objectives of, the Systematic Review clearly stated?

Yes

Is the statistical analysis and its interpretation appropriate?

Not applicable

Are sufficient details of the methods and analysis provided to allow replication by others?

Yes

Are the conclusions drawn adequately supported by the results presented in the review?

Yes

Reviewer Expertise:

Food chemistry - analysis of fried potato product for reducing sugars, amino acids, and acrylamide

I confirm that I have read this submission and believe that I have an appropriate level of expertise to confirm that it is of an acceptable scientific standard.

# Reviewer report: Reviewer response for version 1

**DOI:** 10.5256/f1000research.154356.r221641

**Published:** 2023-12-18

**Authors:** Traiphop Phahom

**Affiliations:** 1 School of Food Technology, Institute of Agricultural Technology, Suranaree University of Technology, Nakhon Ratchasima, Thailand

The manuscript is well constructed. The content is academically sound and interesting for the production of deep-fried foods.

 However, there are some points that need to be considered.

 1. The writing is required to be clear and concise because there are a number of compound and complex sentences used in this manuscript, some of which make the sentences unclear and less concise.

 2. Misspellings are found in the manuscript so, the authors are required to check them.

 3. Some references are required for the topic "Applications and inventors" (paragraphs 2,3,5 and 6).

 4. The authors did not mention GC-MS analysis in the result section, but it appears in the conclusion part. Therefore, the authors are required to review and revise.

Are the rationale for, and objectives of, the Systematic Review clearly stated?

Yes

Is the statistical analysis and its interpretation appropriate?

I cannot comment. A qualified statistician is required.

Are sufficient details of the methods and analysis provided to allow replication by others?

Yes

Are the conclusions drawn adequately supported by the results presented in the review?

Yes

Reviewer Expertise:

Food processing and analysis.

I confirm that I have read this submission and believe that I have an appropriate level of expertise to confirm that it is of an acceptable scientific standard.
